# Sex differences in contaminant concentrations of fish: a synthesis

**DOI:** 10.1186/s13293-016-0090-x

**Published:** 2016-09-02

**Authors:** Charles P. Madenjian, Richard R. Rediske, David P. Krabbenhoft, Martin A. Stapanian, Sergei M. Chernyak, James P. O’Keefe

**Affiliations:** 1 U. S. Geological Survey, Great Lakes Science Center, 1451 Green Road, Ann Arbor, MI 48105 USA; 2 Annis Water Resources Institute, Grand Valley State University, 740 West Shoreline Drive, Muskegon, MI 49441 USA; 3 U. S. Geological Survey, Wisconsin Water Science Center, 8505 Research Way, Middleton, WI 53562 USA; 4 U. S. Geological Survey, Great Lakes Science Center, Lake Erie Biological Station, 6100 Columbus Avenue, Sandusky, OH 44870 USA; 5 University of Michigan, School of Public Health, 1420 Washington Heights, Ann Arbor, MI 48109 USA; 6 Bureau of Laboratories, Michigan Department of Health and Human Services, 3350 North Martin Luther King Jr. Boulevard, Lansing, MI 48906 USA

**Keywords:** Androgens, Bioenergetics models, Gonadosomatic index, Gross growth efficiency, Hg-elimination rates, Laboratory mice, Teleost fishes, Testosterone, Vertebrates

## Abstract

A comparison of whole-fish polychlorinated biphenyl (PCB) and total mercury (Hg) concentrations in mature males with those in mature females may provide insights into sex differences in behavior, metabolism, and other physiological processes. In eight species of fish, we observed that males exceeded females in whole-fish PCB concentration by 17 to 43 %. Based on results from hypothesis testing, we concluded that these sex differences were most likely primarily driven by a higher rate of energy expenditure, stemming from higher resting metabolic rate (or standard metabolic rate (SMR)) and higher swimming activity, in males compared with females. A higher rate of energy expenditure led to a higher rate of food consumption, which, in turn, resulted in a higher rate of PCB accumulation. For two fish species, the growth dilution effect also made a substantial contribution to the sex difference in PCB concentrations, although the higher energy expenditure rate for males was still the primary driver. Hg concentration data were available for five of the eight species. For four of these five species, the ratio of PCB concentration in males to PCB concentration in females was substantially greater than the ratio of Hg concentration in males to Hg concentration in females. In sea lamprey (*Petromyzon marinus*), a very primitive fish, the two ratios were nearly identical. The most plausible explanation for this pattern was that certain androgens, such as testosterone and 11-ketotestosterone, enhanced Hg-elimination rate in males. In contrast, long-term elimination of PCBs is negligible for both sexes. According to this explanation, males not only ingest Hg at a higher rate than females but also eliminate Hg at a higher rate than females, in fish species other than sea lamprey. Male sea lamprey do not possess either of the above-specified androgens. These apparent sex differences in SMRs, activities, and Hg-elimination rates in teleost fishes may also apply, to some degree, to higher vertebrates including humans. Our synthesis findings will be useful in (1) developing sex-specific bioenergetics models for fish, (2) developing sex-specific risk assessment models for exposure of humans and wildlife to contaminants, and (3) refining Hg mass balance models for fish and higher vertebrates.

## Background

In walleye (*Sander vitreus*), lake trout (*Salvelinus namaycush*), and coho salmon (*Oncorhynchus kisutch*) populations, mature males were higher in whole-fish polychlorinated biphenyl (PCB) concentration than mature females by 19 to 34 % [1]. By evaluating four hypotheses to explain the higher whole-fish PCB concentrations in males compared with females in these three fish populations, Madenjian [1] developed a synthesis on the effect of sex on PCB concentrations in fish. These hypotheses included (1) females experienced a drastic reduction in PCB concentration due to release of eggs at spawning and therefore had lower PCB concentrations than males, (2) a difference in habitat utilization between the sexes caused the difference in PCB concentrations between the sexes, (3) females grew faster than males and therefore the growth dilution effect led to a sex difference in PCB concentrations, and (4) males expended energy, via a higher resting metabolic rate (or standard metabolic rate (SMR)) and higher swimming activity, and consequently fed at a higher rate than females, and therefore accumulated PCBs at a higher rate. The main conclusion from the synthesis was that, for most fish populations, the primary driver for the observed sex difference in PCB concentrations was a higher rate of energy expenditure for males [1]. A higher rate of energy expenditure in males would result in a higher rate of food consumption by males, and a consequence of this higher rate of food consumption by males would be a greater rate of PCB accumulation in males. In addition, when the growth rates varied considerably between the sexes, the growth dilution effect could also make a substantial contribution to the observed difference in PCB concentrations between the sexes, although the sex difference in energy expenditure rates was still the primary driver of the observed difference in PCB concentrations between the sexes. The work by Madenjian [1] represented the first published synthesis on the effect of sex on PCB concentrations in fish.

Although higher PCB concentrations in males compared with females has often been attributed to the PCB concentration of females undergoing a drastic decrease due to release of eggs at spawning, the available evidence to support this hypothesis was weak. Based on determinations of PCB concentrations in the muscle tissue of female and male northern pike (*Esox lucius*) and in eggs from female northern pike from a Scandinavian lake, Larsson et al. [2] postulated that higher PCB concentrations in the muscle tissue of males compared with females was due to females releasing eggs at spawning. Assuming that the PCB concentration for all of the somatic tissue in the northern pike was equal to the muscle PCB concentration, Madenjian [1] estimated that PCB concentration of female northern pike would be expected to decrease by 80 % immediately after spawning due to release of eggs. However, this value was an outlier compared with the expected percent change in PCB concentration immediately after spawning due to release of eggs of females from other fish populations. For these other fish populations, PCB concentrations in the ovaries and all of the somatic tissue (rather than just the muscle tissue) were determined, and the expected percent changes in PCB concentration due to release of eggs at spawning were modest, ranging from −4.8 to +13.5 %. For most populations, PCB concentration of the females was expected to slightly increase immediately after spawning due to release of eggs. This slight increase in PCB concentration of females could, in no way, account for the higher PCB concentration observed in males. In addition, the coho salmon used in the study cited by Madenjian [1] were caught prior to spawning. Coho salmon are semelparous in that they spawn only once during their lifetime and then die soon thereafter [3]. Because the coho salmon used in the study had never spawned, release of eggs at spawning could not possibly account for the higher PCB concentrations observed in males compared with females. Madenjian [1] concluded that release of eggs at spawning did not contribute to the observed sex difference in PCB concentrations in most fish populations.

For a sex difference in habitat utilization to cause a sex difference in PCB concentrations, two conditions must be met: (1) the ecosystem inhabited by the fish population must contain a PCB “hot spot”, such that PCB sediment concentration in the hot spot area is several orders of magnitude higher than PCB sediment concentrations in other areas of the ecosystem, and (2) fish of one sex inhabit areas in the vicinity of the hot spot, where PCB concentrations of the prey are elevated, while fish of the opposite sex inhabit other areas where prey PCB concentrations are relatively low [1]. This hot spot effect is not realized if one or both of these conditions are not met. We would expect that both of these conditions are not met for most fish populations. Thus, a sex difference in habitat utilization would be expected to cause a sex difference in PCB concentrations in relatively few fish populations [1]. Nonetheless, a hot spot effect appears to have been realized for a Saginaw Bay walleye population and a Hudson River striped bass (*Morone saxatilis*) population [4–6].

Growth dilution made a substantial contribution to the difference in PCB concentrations between the sexes of South Manistique Lake walleye [7], but had a negligible effect on the sex difference in PCB concentrations for the coho salmon and lake trout populations [3, 8]. For South Manistique Lake walleye, females were more than 40 % larger, by weight, than males over ages 6–8 [7]. In contrast, growth rates did not appreciably vary between the sexes in the lake trout and coho salmon populations. The growth dilution effect was quantified by assuming that SMR and activity rate did not vary between the sexes, and then fitting fish bioenergetics models to the growth trajectories to estimate food consumption for both sexes [1]. PCB concentration is inversely proportional to gross growth efficiency (GGE), which is defined as growth divided by the amount of food needed to attain that growth. If males had a 20 % greater PCB concentration than females, and the growth dilution effect explained all of the difference in PCB concentrations between the sexes, then bioenergetics modeling (under the assumption that SMR and activity rate did not vary between the sexes) would reveal that females had a 20 % greater GGE than males. Although the growth dilution effect accounted for about a third of the observed difference in PCB concentrations between the sexes of South Manistique Lake walleye, other factors would need to be invoked to explain the bulk of the difference.

Few studies have directly addressed sex differences in SMR and swimming activity in fish, but results from these studies supported the contention that males expended energy at a higher rate than females [1]. SMR of brown trout (*Salmo trutta*) was estimated to be 14 % higher in males than in females [9], and SMR of the deep-sea fish *Sebastolobus altivelis* was estimated to be 16 % higher in males than in females [10]. Activity by males was substantially greater than activity by females in both Atlantic salmon (*Salmo salar*) and brook trout (*Salvelinus fontinalis*) [11–13]. Madenjian [1] concluded that males expended energy at a higher rate than females in most or all fish populations. Consequently, PCB concentration in males would exceed PCB concentration in females in most fish populations.

Given that both PCBs and total mercury (Hg) are reliable tracers of food consumption by fish [14–16], the ratio of whole-fish PCB concentration in males to whole-fish PCB concentration in females should equal the ratio of whole-fish Hg concentration in males to whole-fish Hg concentration in females. Nearly all of the PCBs and Hg accumulated by fish is believed to enter the bodies of the fish via dietary intake [14–16]. Thus, the relative difference in PCB concentrations between the sexes should equal the relative difference in Hg concentrations between the sexes, all other factors being equal.

The goal of our study was to formulate a new synthesis on sex differences in contaminant (PCBs and Hg) concentrations of fish. More than 5 years have passed since the Madenjian [1] synthesis, which was based on whole-fish PCB determinations for just three species of fish. The four hypotheses identified in the first synthesis need to be reevaluated, and conclusions from the first synthesis need to be critically assessed based on new whole-fish PCB data. In addition, since the time of the first synthesis, the relative sex difference in PCB concentrations has been compared with the relative sex difference in Hg concentrations for several species of fish [17–21]. However, certain sets of statistical comparisons of the effect of sex on PCB concentration with the effect of sex on Hg concentration have yet to be completed. Our specific objectives included (1) reevaluate the four hypotheses identified in the Madenjian [1] synthesis, (2) reevaluate the conclusions from the Madenjian [1] synthesis, (3) complete additional statistical comparisons of the effect of sex on PCB concentration with the effect of sex on Hg concentration, (4) reconcile any differences between PCBs and Hg with regard to sex differences in contaminant concentrations, (5) briefly discuss the implications from our synthesis on sex differences in accumulation of contaminants by fish for higher vertebrates, and (6) identify new research directions in the study of sex differences in contaminant accumulation in fish. All of the fish that we used to develop this synthesis were handled according to the regulations established under the Animal Welfare Act, which is a set of animal welfare guidelines abided by U. S. Federal Government researchers. Procedures followed in our research were approved by the U. S. Geological Survey Great Lakes Science Center (study plan ND00GA0.01).

## Sex difference in PCB concentrations

In light of new research, the conclusion drawn in the first synthesis that males would exceed females in PCB concentration in most fish populations, primarily as a consequence of higher energy expenditure rate in males, appeared to be sound. Males exceeded females in PCB concentration by 17 to 43 % in populations of burbot (*Lota lota*), sea lamprey (*Petromyzon marinus*), cisco (*Coregonus artedi*), lake whitefish (*Coregonus clupeaformis*), and summer flounder (*Paralichthys dentatus*) (Table 1). Thus, in all eight species, whole-fish PCB concentration in males was greater than whole-fish PCB concentration in females. After consideration of all four abovementioned hypotheses, the most likely primary driver of the sex difference in PCB concentrations was consistently identified as a higher rate of energy expenditure rate in males compared with females [3, 7, 8, 22–27]. The pattern was persistent, regardless of whether the fish species were freshwater or marine. In a recent study, swimming activity by male sea lamprey was found to be 27 to 69 % greater than swimming activity by female sea lamprey [23], providing further corroboration for the contention that male fish are more active than female fish.

**Table 1:** Estimates of the ratio of whole-fish PCB concentration in males to whole-fish PCB concentration in females (PCB_**♂**_:PCB_**♀**_) and the ratio of whole-fish Hg concentration in males to whole-fish Hg concentration in females (Hg_**♂**_:Hg_**♀**_)

Species	Population	PCB_**♂**_:PCB_**♀**_	Hg_**♂**_:Hg_**♀**_	References
Walleye	South Manistique Lake	1.34	NA	7
Lake trout	Lake Ontario	1.22	1.08	8, 17
Coho salmon	Lake Michigan	1.19	NA	3
Burbot	Lake Erie, Great Slave Lake	1.29	0.82	19, 22, 24
Sea lamprey	Lake Huron	1.17	1.16	18, 23
Cisco	Lake Superior	1.43	NA	25
Lake whitefish	Lake Huron	1.34	0.91	20, 26
Summer flounder	New Jersey coast	1.43	0.98	21, 27

PCB_**♂**_:PCB_**♀**_ was equal to 1.28 and 1.30 for Lake Erie (ages 6–13) burbot and for Great Slave Lake burbot, respectively, and the average between the two lakes is reported in this table. Hg_**♂**_:Hg_**♀**_ for burbot was estimated by pooling data from Lake Erie and Great Slave Lake

NA not available

In light of new findings, the conclusion drawn in the first synthesis that the growth dilution effect could substantially contribute to the observed sex difference in some cases, but not serve as the primary driver of the difference, appeared to be sound. Female summer flounder grew sufficiently faster than male summer flounder such that by age 8, females were nearly double the size, by weight, of males [27]. In contrast, the sex differences in growth rates were modest for burbot, sea lamprey, cisco, and lake whitefish [22–26]. In fact, growth rates of burbot from Great Slave Lake did not appreciably vary between the sexes [24]. Bioenergetics modeling results revealed that the growth dilution effect could account for male summer flounder being 18.5 % higher in PCB concentration than female summer flounder, but male summer flounder were observed to be 43 % higher in PCB concentration than female summer flounder [27]. Thus, although the growth dilution effect made a substantial contribution (18.5/43 or 43 %) to the observed sex difference in PCB concentrations, the primary driver of the sex difference was not the growth dilution effect. Rather, the most likely primary driver for the sex difference in PCB concentrations was a higher energy expenditure rate for males [27]. Contributions of the growth dilution effect toward the observed sex difference in PCB concentrations were considerably less for the other four species than that for summer flounder. The growth dilution effect accounted for 0.5/17 or 3 % of the observed sex difference in PCB concentrations in sea lamprey [23], 3/43 or 7 % of the observed sex difference in PCB concentrations in cisco [25], and 0.7/34 or 2 % of the observed sex difference in PCB concentrations in lake whitefish [26]. For burbot, the contribution of the growth dilution effect toward the observed sex difference was negligible [24]. Thus, growth dilution had a substantial effect in only two of eight fish species.

Given the new findings, the conclusion drawn in the first synthesis that the hot spot effect would be realized in relatively few fish populations remained valid. A hot spot effect did not appear to be operating in younger burbot (ages 6–13) in Lake Erie, burbot in Great Slave Lake, sea lamprey from Lake Huron, cisco from Lake Superior, lake whitefish from northern Lake Huron, or summer flounder off the New Jersey coast [22–27]. Evidence for a hot spot effect was apparent only for a population of older (ages 14–17) burbot from Lake Erie [22]. In this case, the suspected PCB hot spot was the mouth of the Ashtabula River, a tributary to Lake Erie [28]. A substantial difference in PCB congener distributions between the sexes typically accompanies a hot spot effect [29, 30]. PCB congener distributions varied dramatically between the sexes of these older (ages 14–17) burbot from Lake Erie, whereas PCB congener distributions varied little between the sexes of younger (ages 6–13) burbot from Lake Erie [29]. Likewise, a sex difference in PCB congener distributions was not detected in burbot from Great Slave Lake or in summer flounder from New Jersey coastal waters, two fish populations in which a hot spot effect was not realized [27, 30]. In sum, a hot spot effect appeared to occur in relatively few fish populations.

**Fig. 1:**
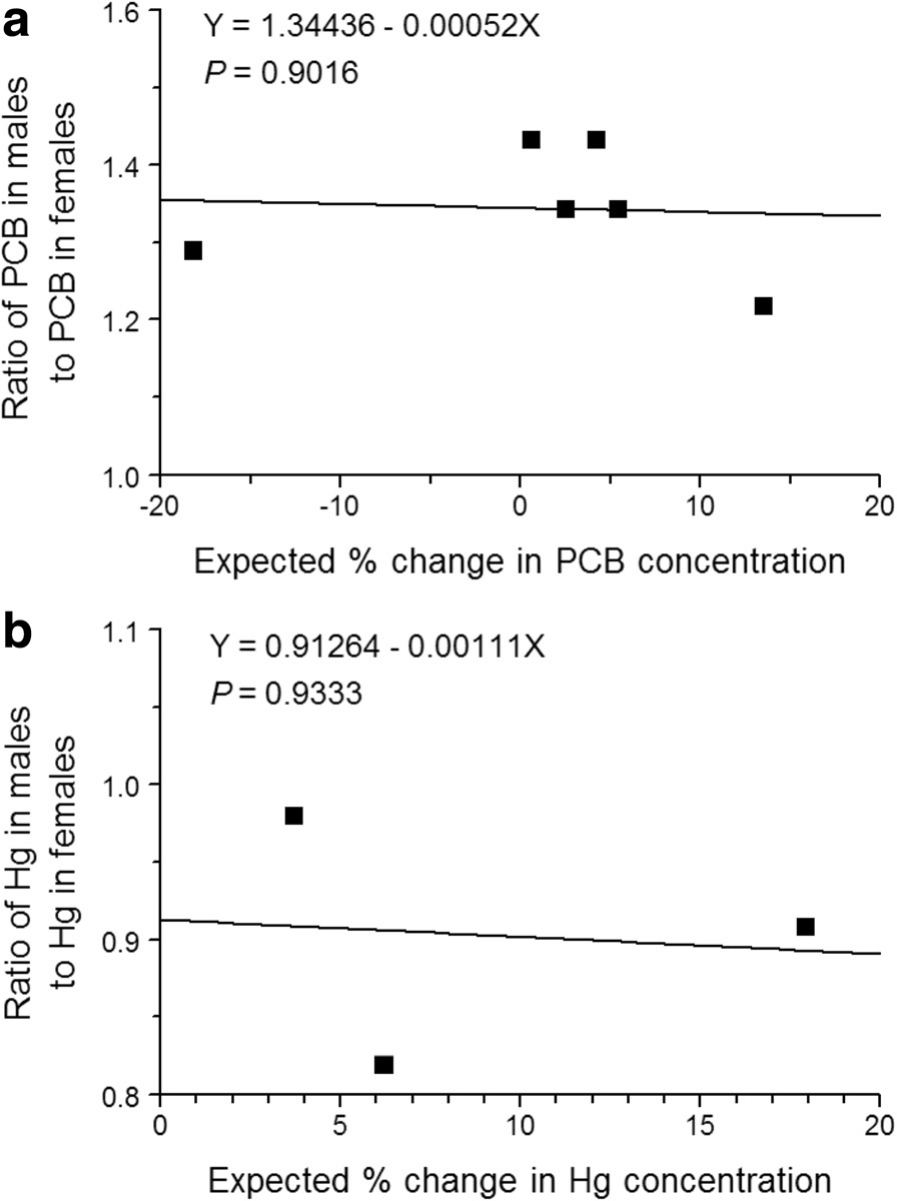
**a** Ratio of whole-fish PCB concentration in males to whole-fish PCB concentration in females as a function of expected percent change in whole-fish PCB concentration of female fish immediately after spawning due to release of eggs. **b** Ratio of whole-fish Hg concentration in males to whole-fish Hg concentration in females as a function of expected percent change in whole-fish Hg concentration of female fish immediately after spawning due to release of eggs. Fitted regression line is shown. Attained significance level, *P*, of the *F* test for significance of the regression line is also shown. Data taken from Tables 1 and 2

Because the postulations from the Larsson et al. [2] study were questionable, and in light of additional evidence, we conclude that the immediate change in PCB concentration in females due to release of eggs at spawning apparently has a negligible effect on the long-term trajectory of PCB concentration in adult females in the overwhelming majority of fish populations. Accordingly, the long-term effect of the release of eggs at spawning on the sex difference in PCB concentrations is negligible in the overwhelming majority of fish populations. The assumption that muscle PCB concentration was equal to the PCB concentration of all of the somatic tissue in the female northern pike was likely invalid, based on results from previous studies. Northern pike is a relatively lean fish [31], as is yellow perch (*Perca flavescens*) [32]. In a mixed-sex sample of yellow perch, whole-fish PCB concentration was 10 times higher than skin-off fillet PCB concentration [33]. Further, egg PCB concentration has been documented to be only 26 % greater than somatic tissue PCB concentration in yellow perch [32]. Collectively, these results suggested that somatic tissue PCB concentration in northern pike was underestimated by assuming that muscle PCB concentration was equal to PCB concentration of all of the somatic tissue. Consequently, the magnitude of the expected percent change in PCB concentration immediately after spawning due to release of eggs was overestimated. For cases in which PCB concentrations in all of the somatic tissue and in the ovaries were determined, the expected percent change in PCB concentration immediately after spawning due to release of eggs ranged from −18.2 to +13.5 % (Table 2). Variation in the expected percent change in PCB concentration due to release of eggs did not have a significant effect on the ratio of PCB concentration in males to PCB concentration in females (Fig. 1a). We hypothesize that the modest changes in PCB concentration of females due to release of eggs at spawning represent minor perturbations in the long-term trajectory of whole-fish PCB concentration of the females. Within a matter of a few weeks after spawning, we would expect the PCB concentration of the females to return to the path of the long-term trajectory of whole-fish PCB concentration. All of the PCB determinations reported in Table 1, except for those of burbot from Lake Erie, were on fish sampled from spawning aggregations prior to spawning [3, 7, 8, 23–27]. Burbot from Lake Erie were caught in August and September, roughly 6 or 7 months after spawning time in February [22]. Further, sea lamprey, like coho salmon, is a semelparous species [34]. Because the sea lamprey were caught prior to spawning, the sea lamprey used in the Madenjian et al. [23] study had never spawned prior to capture. Release of eggs at spawning could not possibly account for the observed sex difference in sea lamprey PCB concentrations. Yet, male sea lamprey were higher in PCB concentration than female sea lamprey, just as was the case for the other seven fish species listed in Table 1.

**Table 2:** Estimates of the expected percent change in whole-fish PCB concentration of female fish immediately after spawning due to release of eggs (Δ PCB) and the expected percent change in whole-fish Hg concentration of female fish immediately after spawning due to release of eggs (Δ Hg)

Species	Population	Δ PCB	Δ Hg	References
Rainbow trout	Lake Ontario	+9.1	+15.3	32
White sucker	Lake Ontario	+9.1	+16.0	32
White bass	Lake Erie	-0.5	+10.9	32
Smallmouth bass	Lake Erie	−2.2	+5.5	32
Yellow perch	Lake Erie	−4.8	+22.4	32
Walleye	Saginaw Bay	+4.5	NA	4
Walleye	South Manistique Lake	+5.4	NA	7
Lake trout	Lake Michigan	+13.5	NA	8
Burbot	Great Slave Lake	−18.2	+6.8	19, 24
Cisco	Lake Superior	+4.2	NA	25
Lake whitefish	Lake Huron	+2.5	+17.9	20, 26
Summer flounder	New Jersey coast	+0.6	+3.7	21, 27

NA not available

Although lipid concentration tends to show a positive correlation with PCB concentration, lipid concentration does not control PCB accumulation in fish [35–39]. Rather, food consumption controls PCB accumulation in fish. Clearly, the whole-fish contaminant results that we report in this synthesis paper do not support the assertion that the sex difference in PCB concentrations was related to a sex difference in lipid concentrations. In South Manistique Lake walleye, Great Slave Lake burbot, Lake Huron sea lamprey, and Lake Superior cisco, females averaged a slightly higher lipid concentration compared with males [7, 23–25]. Conversely, in Lake Ontario lake trout, Lake Michigan coho salmon, Lake Erie burbot, and summer flounder from the New Jersey coast, males were slightly higher in lipid concentration than females, on average [3, 8, 22, 27]. Yet, PCB concentrations were consistently higher in males than in females in all eight fish species (Table 1).

Because mean age of females was very similar to, or identical to, mean age of males in all cases (Table 3), a sex difference in fish ages within a given fish population did not contribute to any of the observed sex differences in whole-fish PCB concentrations. For the case of Lake Ontario lake trout, PCB concentration significantly increased with increasing age [8]. In other cases, PCB concentration trended neither upward nor downward with increasing age [7, 22, 24, 26, 27]. Analysis of covariance (ANCOVA), with age as a covariate, was used to estimate the sex difference in whole-fish PCB concentrations for Lake Ontario lake trout [8]. Following this ANCOVA approach, the ratio of whole-fish PCB concentration in males to whole-fish PCB concentration in females was estimated at a lake trout age of 5.7 years, which was the grand mean of age for both sexes pooled. An inclusion of age as a covariate in any of the statistical analyses used to estimate the sex difference in whole-fish PCB concentrations for the other fish populations was unwarranted [3, 7, 22–27]. All of the sampled coho salmon, sea lamprey, and cisco were ages 2, 1, and 7 years, respectively [3, 23, 25].

**Table 3:** Mean age, in years, of the females and the males used to estimate the sex difference in whole-fish PCB concentrations

Species	Population	Mean age of females	Mean age of males	Reference
Walleye	South Manistique Lake	5.7 (0.3)	5.1 (0.3)	7
Lake trout	Lake Ontario	5.5 (0.1)	5.8 (0.1)	8
Coho salmon	Lake Michigan	2.0 (0.0)	2.0 (0.0)	3
Burbot	Lake Erie	9.8 (0.9)	9.5 (1.0)	22
Burbot	Great Slave Lake	11.0 (0.7)	11.5 (0.7)	24
Sea lamprey	Lake Huron	1.0 (0.0)	1.0 (0.0)	23
Cisco	Lake Superior	7.0 (0.0)	7.0 (0.0)	25
Lake whitefish	Lake Huron	10.4 (0.4)	10.9 (0.4)	26
Summer flounder	New Jersey coast	3.7 (0.2)	4.2 (0.2)	27

Standard error of the mean enclosed within parentheses

For sea lamprey, age refers to the presumed number of years that the sea lamprey lived in the lake feeding on the blood of other fish before ascending the river to spawn

## Sex difference in Hg concentrations

Males exceeded females in whole-fish Hg concentration in some fish species, whereas females had higher whole-fish Hg concentrations than males in other fish species (Table 1). Of the eight fish species listed in Table 1, Hg concentration data were available for five species. For these five species, Hg concentrations were determined for the same fish used for PCB determinations. That is, for each fish, two jars of homogenates were filled; one jar was for PCB determination and the other jar was for Hg determination. The ratio of Hg concentration in males to Hg concentration in females ranged from 0.82 to 1.16 (Table 1).

Expected percent change in Hg concentration of females immediately after spawning due to release of eggs ranged from +3.7 to +22.4 (Table 2). These expected percent changes were estimated by determining Hg concentrations in both the eggs and the somatic tissue of females caught in the wild. Based on the available data, the expected percent change in whole-fish Hg concentration of females due to release of eggs at spawning was not significantly related to the ratio of whole-fish Hg concentration in males to whole-fish Hg concentration in females (Fig. 1b). Thus, just as for PCBs, we hypothesize that the modest changes in Hg concentration of females immediately after spawning due to release of eggs represent minor perturbations in the long-term trajectory of Hg concentration for females in the overwhelming majority of fish populations. Further, these minor perturbations have a negligible effect on the long-term trajectory of Hg concentration in females.

A hot spot effect for Hg has not been documented in any fish population in the Laurentian Great Lakes, Great Slave Lake, or New Jersey coastal waters, and therefore, such an effect would not be expected to make any contribution to the observed sex differences in Hg concentrations listed in Table 1. Further, evidence for a sex difference in habitat utilization was lacking for the Lake Ontario lake trout population, Lake Huron sea lamprey population, Great Slave Lake burbot population, and Lake Huron lake whitefish population [17–20]. Thus, factors other than a hot spot effect were likely responsible for the observed sex differences in Hg concentrations.

## Sex difference in PCB concentrations versus sex difference in Hg concentrations

For species other than sea lamprey, the ratio of Hg concentration in males to Hg concentration in females was substantially lower than the ratio of PCB concentration in males to PCB concentration in females (Table 1). In sea lamprey, the two ratios were very similar, with males exceeding females by 17 and 16 % in PCB concentration and Hg concentration, respectively. The considerable difference between these two ratios in lake trout, burbot, lake whitefish, and summer flounder was unexpected, because PCBs and Hg are considered reliable tracers of food consumption by fish [14–16]. Clearly, PCBs are processed in a different manner than the manner in which Hg is processed by teleost fishes, including lake trout, burbot, lake whitefish, and summer flounder. From an evolutionary standpoint, teleost fishes (superclass Osteichthyes, class Actinopterygii, infraclass Teleostei), also referred to as modern bony fishes, represent the most advanced infraclass of fishes [40]. In stark contrast, the sea lamprey, a jawless and cartilaginous fish in the lamprey class (superclass Agnatha, class Petromyzontida), is considered one the most primitive of all fishes [40].

**Fig. 2:**
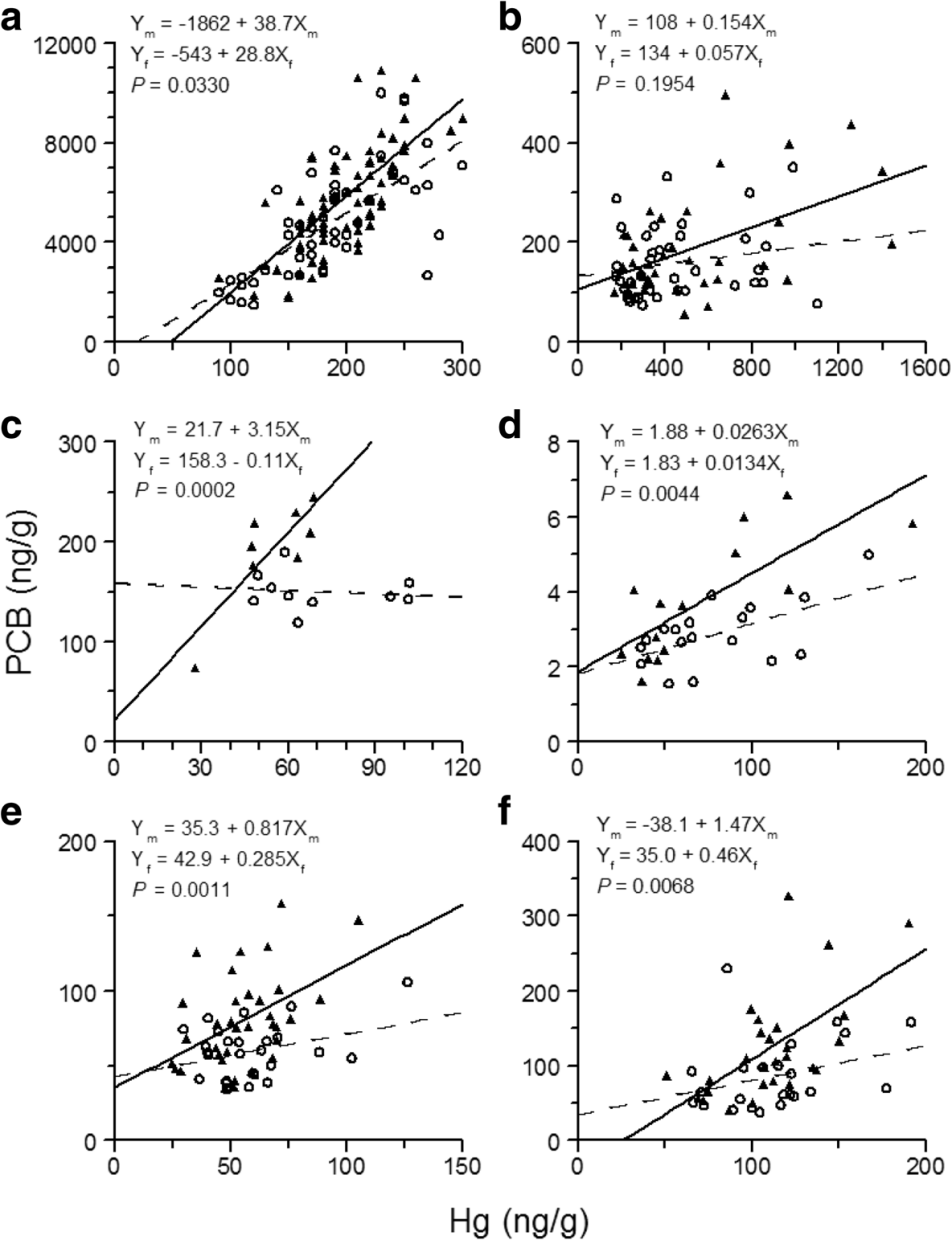
PCB concentration as a function of Hg concentration for both males (*solid triangles*) and females (*hollow circles*). Also shown are fitted regression lines for males (*solid lines*) and females (*dashed lines*). In each panel, the upper regression equation is for males (subscript *m* denotes males) and the lower regression equation is for females (subscript f denotes females). In each panel, the attained significance level, *P*, of the *F* test for determining whether the regression line for males is significantly different from the regression line for females is also shown. Data taken from (**a**) lake trout from Lake Ontario [8, 17]; (**b**) sea lamprey from Lake Huron [18, 23]; (**c**) burbot (ages 6–13) from Lake Erie [19, 22]; (**d**) burbot from Great Slave Lake [19, 24]; (**e**) lake whitefish from Lake Huron [20, 26]; and (**f**) summer flounder from New Jersey coast [21, 27]

The effect of sex on Hg concentration was significantly different from the effect of sex on PCB concentration in lake trout, burbot, lake whitefish, and summer flounder, but not so in sea lamprey. The regression line of PCB concentration as a linear function of Hg concentration for females was significantly different from that for males in the four abovementioned teleost fishes (Fig. 2a, c–f), but no difference between the line for females and the line for males was detected in sea lamprey (Fig. 2b). Moreover, if the difference between the ratio of PCB concentration in males to PCB concentration in females and the ratio of Hg concentration in males to Hg concentration in females is treated as an independent observation for each of the four teleost species, then the PCB concentration ratio is significantly greater than the Hg concentration ratio for these four teleost fishes (*t* test for paired comparisons: *t* = 4.78; df = 3; *P* = 0.0174).

The most plausible explanation for the significant difference between the effect of sex on PCB concentrations and the effect of sex on Hg concentrations in the teleost fishes was that males eliminated Hg at a substantially faster rate than females, whereas long-term elimination of PCBs was negligible for both sexes of fish. Although the rate at which fish eliminate Hg from their bodies is slow, Hg-elimination rates are measurable [41–43]. In contrast, long-term decreases in the PCB body burden of fish are undetectable. The PCB body burden of a fish refers to the weight of PCBs contained within the fish. In four experiments designed to measure PCB elimination rate, PCB body burden in fish showed no further decrease over a period of months following an initial phase (several days in duration), during which PCB body burden did decrease immediately after dosing of fish [44]. Thus, long-term elimination of PCBs by fish can be considered negligible for both sexes. A faster rate of elimination of Hg by males compared with that by females in teleost fishes can account for the ratio of PCB concentration in males to PCB concentration in females exceeding the ratio of Hg concentration in males to Hg concentration in females. Summarizing the formulation of our synthesis to this point, males ingest both PCBs and Hg at a higher rate than females, primarily due to a higher rate of energy expenditure. However, in teleost fishes, males also eliminate Hg at a considerably faster rate than females while long-term elimination of PCBs is negligible for both sexes. Thus, this disparity in sex differences in elimination rates between the two contaminant types (PCBs and Hg) explains the ratio of PCB concentration in males to PCB concentration in females exceeding the ratio of Hg concentration in males to Hg concentration in females in teleost fishes.

Results from several studies supported the contentions that Hg-elimination rate for males is substantially faster than Hg-elimination rate for females in teleost fishes and higher vertebrates, and that this sex difference in Hg-elimination rates was attributable to certain androgens. As juveniles, rates of eliminating Hg via excretion of urine did not vary between the sexes of laboratory mice (*Mus musculus*) [45]. However, when the mice approached maturity, the Hg-elimination rate for males increased to a level that was approximately three times higher than that for females, and this sex difference in Hg-elimination rates was maintained throughout adulthood. Further experimentation on laboratory mice revealed that the Hg-elimination rate via urine excretion was linked to activity of the enzyme γ-glutamyltranspeptidase (γ-GTP) in the kidneys, and that testosterone enhanced γ-GTP activity in the kidneys [46, 47]. Moreover, γ-GTP activity in the kidneys of males approaching maturity decreased to the same level as that for females approaching maturity when the males were castrated [48]. Furthermore, when females were injected with testosterone, the γ-GTP activity in their kidneys increased to the same level as that for mature males. These laboratory experiments provided compelling evidence that testosterone enhanced Hg-elimination rate [49]. Adult male northern pike were estimated to eliminate Hg from their bodies at more than double the rate of that for adult female northern pike, based on tracking isotope-enriched Hg concentrations over a 3-year period [50]. Because testosterone found in teleost fishes is identical in chemical structure to testosterone found in mammals and other higher vertebrates [51–54], Madenjian et al. [50] proposed that the higher Hg-elimination rate for male northern pike was due to enhancement of Hg-elimination rate by certain androgens, including testosterone. Enhancement of Hg-elimination rate in male teleost fish may also be attributable to 11-ketotestosterone, an androgen very similar in chemical structure to testosterone and found in all teleost fishes [19–21, 50, 53].

However, testosterone and 11-ketotestosterone are not found in sea lamprey [55]. Rather, the androgens found in sea lamprey are substantially different in chemical structure than androgens found in other vertebrates [55]. Perhaps the androgens found in sea lamprey do not enhance Hg-elimination rate in males [18]. If so, then this would explain the ratio of PCB concentration in males to PCB concentration in females nearly equaling the ratio of Hg concentration in males to Hg concentration in females in sea lamprey (Table 1). Without enhancement of Hg-elimination rate for males by androgens, Hg-elimination rates would not vary between the sexes, and therefore, the ratio of PCB concentration in males to PCB concentration in females would equal the ratio of Hg concentration in males to Hg concentration in females.

We contend that the difference between the ratio of PCB concentration in males to PCB concentration in females and the ratio of Hg concentration in males to Hg concentration in females is primarily determined by the sex difference in Hg-elimination rates. The difference between the two abovementioned ratios should increase with an increasing sex difference in Hg-elimination rates. A corollary to our contention is that Hg-elimination rate for males varies substantially from one species to another. This corollary is corroborated by two pieces of evidence. First, Kostyniak [56] demonstrated that Hg-elimination rate for males of the CFW strain of laboratory mice was five times higher than that of males of the CBA/J strain of laboratory mice. Second, estimates of Hg-elimination rates from laboratory experiments indicated that lake whitefish were capable of eliminating Hg from their bodies at a rate nearly three times faster than that for lake trout [20, 57, 58]. These Hg-elimination rates were derived from mixed-sex fish populations held in laboratory tanks. Further, we propose that the gonadosomatic index (GSI), which is equal to the proportion of the total weight of the fish represented by the gonads, of males influences the sex difference in Hg-elimination rates, which in turn influences the sex difference in Hg concentrations relative to the sex difference in PCB concentrations. Thus, we hypothesize that the concentration of androgens like testosterone and 11-ketotestosterone increases with increasing GSI of the males, and that Hg-elimination rate increases with increasing concentration of these androgens. In support of this hypothesis, several studies on mammals and birds have shown that testosterone concentration is positively correlated with GSI of males. Dixson [59] showed that, in primates, the interspecific variation in testosterone levels of males can be attributed, in part, to interspecific variation in the GSIs of males, and that testosterone levels of males were positively related to the GSIs of the males. Similarly, Garamszegi et al. [60] documented that, based on a survey of >100 species of birds, peak testosterone levels of male birds were positively related to their GSIs. Preston et al. [61] found that testosterone levels in male Soay sheep (*Ovis aries*) were positively correlated with testes size. In all three studies, the researchers concluded that increased GSI of males tended to lead to increased testosterone concentration in males.

Although the difference between the ratio of PCB concentration in males to PCB concentration in females and the ratio of Hg concentration in males to Hg concentration in females appeared to be influenced by GSI of the males, other factors also contributed to the difference between these two ratios. Of the species listed in Table 1, the greatest difference between the two abovementioned ratios was for burbot. Male burbot had a much higher GSI (10.8 %) than that for lake trout (3.1 %), lake whitefish (2.1 %), or summer flounder (1.7 %) [8, 19–21], thereby supporting the contention that GSI contributes to the sex difference in Hg concentrations relative to the sex difference in PCB concentrations. However, the GSI for male lake trout was slightly higher than that for male lake whitefish or male summer flounder, yet the difference between the ratio of PCB concentration in males to PCB concentration in females and the ratio of Hg concentration in males to Hg concentration in females was considerably greater for lake whitefish and summer flounder than for lake trout. Clearly, other factors affected the difference between the ratio of PCB concentration in males to PCB concentration in females and the ratio of Hg concentration in males to Hg concentration in females. The levels of androgens like testosterone and 11-ketotestosterone in males were likely controlled by a suite of factors, with GSI of males being just one of the determinants. If so, then males of some fish species could have relatively high androgen levels, despite having low GSIs. Higher androgen levels in male lake whitefish compared with male lake trout would explain the considerably greater difference between the two abovementioned ratios for lake whitefish than for lake trout. In support of this explanation, Hg-elimination rate for lake whitefish has been shown to be nearly three times higher than that for lake trout [57, 58]. Perhaps Hg-elimination rates of males are regulated by a suite of factors, with androgen concentration being just one of these factors. Research on various strains of mice suggests that Hg-elimination rate may be influenced by glutathione content in the liver and kidneys, and that glutathione content in the liver and kidneys exhibits large variation across strains of laboratory mice [47]. Of course, differences in androgen concentration between strains may be driving these differences in glutathione content in the liver and kidneys between strains. Our findings, in conjunction with research findings on laboratory mice, suggest that Hg-elimination rates of males can vary substantially across species of teleost fishes and higher vertebrates.

## Caveats for studying sex differences in contaminant concentrations of fish

To be absolutely certain that the methods used to investigate sex differences in contaminant concentrations of fish accurately capture these differences on a whole-fish basis, whole-fish determinations of the contaminant concentrations are required. Spatial distributions of contaminants within the bodies of fish may vary between the sexes. Consequently, a sex difference in contaminant concentrations based on determinations in just one part (e.g., muscle or liver tissue) of the fish may not accurately capture the sex difference in whole-fish contaminant concentrations. Nevertheless, for some fish species, determinations of contaminant concentration in a certain portion of the fish can result in a reasonably accurate characterization of the sex difference in whole-fish contaminant concentrations. For example, the relative difference in fillet PCB concentrations between the sexes was very similar to the relative difference in whole-fish PCB concentrations between the sexes in both coho salmon and walleye [3, 7]. In other cases, determinations of contaminant concentration in one part of the fish may result in a grossly inaccurate characterization of the sex difference in whole-fish contaminant concentrations. For example, in lake trout, females can exceed males in fillet PCB concentration, whereas males exceed females in whole-fish PCB concentration [1]. To further illustrate the pitfalls associated with using contaminant determinations in just a part of the fish to infer sex differences in whole-fish contaminant concentrations, consider the study by Pedro et al. [62] on Hg concentrations in the muscle tissue and livers of sea lamprey caught in Portuguese rivers. Hg concentrations in a portion of the trunk muscle and in the livers of 80 sea lamprey were determined. Males were about 10 % higher in muscle tissue Hg concentration than females, whereas females were roughly 60 % greater in liver Hg concentration than males. If the liver was targeted as the portion of the fish representative of the whole fish, then researchers would conclude that females were higher than males in Hg concentration on a whole-fish basis. If a portion of the trunk muscle was targeted as representative of the whole fish, then researchers would conclude that males were higher than females in Hg concentration on a whole-fish basis. The relative difference in whole-fish Hg concentrations between the sexes was probably not accurately captured by either determinations of Hg concentration in a portion of the trunk muscle or determinations of Hg concentration in the liver, because both Drevnick et al. [63] and Madenjian et al. [18] found male sea lamprey to be 15 to 16 % higher, on average, in whole-fish Hg concentration than female sea lamprey. In addition, results from recent research have suggested that determinations of Hg concentration in just a very small (0.5 g) plug of white muscle tissue do not accurately capture the sex difference in whole-fish Hg concentrations of bluegill (*Lepomis macrochirus*) [64].

In general, PCB concentrations in muscle tissue and liver tissue of fish tended to be higher in males than in females. Thus, although determinations of PCB concentrations in just part of a fish may not accurately capture the relative difference in whole-fish PCB concentrations between the sexes in many cases, results from PCB determinations on a part of the fish were in coarse agreement with results from whole-fish PCB determinations with regard to the relative difference in PCB concentrations between the sexes. In all eight species listed in Table 1, males exceeded females in whole-fish PCB concentration. Similarly, Kammann et al. [65] reported higher PCB concentrations in the livers of male dab (*Limanda limanda*), a species of flatfish, compared with female dab from the North Sea. Larsson et al. [2] observed higher PCB concentrations in the muscle tissue of male northern pike compared with female northern pike from a Scandinavian lake. Likewise, males exceeded females in fillet PCB concentrations of both largemouth bass (*Micropterus salmoides*) and spotted bass (*Micropterus punctulatus*) from a southeastern USA reservoir [66]. Fillet PCB concentration of male white perch (*Morone americana*) exceeded that of female white perch from a New Jersey estuary [67]. Moreover, Bodiguel et al. [68] reported higher PCB concentrations in both the muscle tissue and liver tissue of adult male European hake (*Merluccius merluccius*) compared with adult female European hake from the Mediterranean Sea. For most of the walleye populations surveyed in Canada, fillet PCB concentration of males was greater than that of females [69]. One exception to this pattern was the case of channel catfish (*Ictalurus punctatus*) from Lake Logan Martin, a reservoir in southeastern USA, where female channel catfish exceeded male channel catfish in fillet PCB concentration [66]. Rypel et al. [66] attributed the higher PCB concentrations in females to a hot spot effect, because results from a mark-recapture study suggested that female channel catfish spent more time in the vicinity of the PCB hot spot than male channel catfish. In contrast, a hot spot effect was not implicated for the observed sex differences in PCB concentrations of largemouth bass and spotted bass from Lake Logan Martin [66]. For five species of fish other than walleye, Gewurtz et al. [69] failed to detect a significant difference in fillet PCB concentrations between the sexes in most of the fish populations surveyed in Canada. This failure to detect a significant difference was partly due to low sample sizes. This failure may have been also partly due to the ratio of fillet PCB concentration in males to fillet PCB concentration in females being considerably lower than the ratio of whole-fish PCB concentration in males to whole-fish PCB concentration in females.

Results from other studies on sex differences in whole-fish PCB concentrations were in accord with the results presented in Table 1. Johnston et al. [70] documented higher whole-fish PCB concentrations in adult male walleye compared with adult female walleye from Lake Huron, Lake Erie, and Lake Ontario. Similarly, Pääkkönen et al. [71] reported higher whole-fish PCB concentrations in adult male burbot than in adult female burbot from Lake Päijänne in Finland.

Sex differences in muscle Hg concentrations did not appear to follow a clear trend, as males exceeded females in muscle Hg concentration with roughly the same frequency at which females exceeded males in muscle Hg concentration. Thus, although determinations of Hg concentrations in just part of a fish may not accurately capture the relative difference in whole-fish Hg concentrations between the sexes in many cases, results from Hg determinations for muscle tissue were in coarse agreement with results from whole-fish Hg determinations with regard to the relative difference in Hg concentrations between the sexes. In two of the five species listed in Table 1, males exceeded females in whole-fish Hg concentration, whereas females exceeded males in whole-fish Hg concentration in the other three species. Similarly, results from a recent review on fish Hg concentrations showed that males were higher in muscle Hg concentration than females in eight studies, whereas females were higher in muscle Hg concentration than males in seven studies [72].

To study sex differences in contaminant concentrations of fish, we recommend that whole-fish contaminant determinations be made on mature fish caught while the fish are in spawning condition. These fish should be sampled before they spawn, for reasons previously discussed. Ages of fish should be estimated to ensure that the mean age for females is similar to the mean age for males. Growth trajectories for both sexes should be elucidated, so that bioenergetics modeling can be used to quantify the growth dilution effect. At least 25 individuals of each sex should be sampled to ensure that a statistically significant difference in contaminant concentrations between the sexes can be detected [8, 23, 26, 27].

## Implications for higher vertebrates

We hypothesize that the sex differences in SMRs, activities, and Hg-elimination rates to which we have ascribed the sex differences in contaminant concentrations of fish also pertain, at least to some degree, to higher vertebrates. For fish in general, we have concluded that males appear to have a higher SMR than females, and that males appear to exhibit higher swimming activity than females. Higher SMR and swimming activity result in a higher energy expenditure rate, which, in turn, leads to a higher rate of food consumption. Further, for teleost fishes, we have concluded that males appear to eliminate Hg at a substantially faster rate than females, and that this higher Hg-elimination rate was likely attributable to enhancement by certain androgens. In support of our hypothesis, SMR in men has been estimated to be significantly higher than SMR in women. Mcmurray et al. [73] collated SMR data from medical research databases and then performed a comprehensive meta-analysis on the collated data. Depending on the data grouping, SMR of men averaged from 6 to 9 % higher than that of women, and these differences were highly significant [73]. Moreover, in reviewing over 100 studies on physical activity of adolescents and then performing a meta-analysis, Sallis et al. [74] found that the effect of sex was highly significant, with physical activity by adolescent boys consistently exceeding physical activity by adolescent girls. Thus, the sex differences in SMRs and activities that we have inferred for fishes also appear to apply to humans as well. Similarly, in a population of the grey mouse lemur (*Microcebus murinus*), a small nocturnal primate endemic to Madagascar, males were more active than females over the course of the year [75, 76]. Based on trapping and radio tracking grey mouse lemurs during 1995, Schmid [75] concluded that adult males were substantially more active than adult females during the dry season. Schmid and Speakman [76] used doubly labeled water to estimate daily energy expenditure (DEE, in kJ/day) in male and female grey mouse lemurs during both the dry and rainy seasons. In addition, the activity multiplier in each lemur was estimated by dividing the DEE estimate by an estimate of energy expended during a 24-h period via resting metabolism, which was calculated by the product (SMR × lemur weight × 24) and then converted into energy units. The SMR estimate was derived from previously published regression equations of SMR as a function of male grey mouse lemur weight for the dry and rainy seasons; SMR determinations were made on males only. Results showed that DEE of males exceeded DEE of females by 11 %, on average, during the rainy season, even though females were 13 % greater in weight than males. In addition, males averaged a 7 % greater DEE than females during the dry season, despite the females being 29 % greater in weight than the males. On average, activity multiplier of the males exceeded that of the females by 14 and 22 % during the rainy and dry seasons, respectively [76]. The experimental work by Hirayama and Yasutake [45], Yasutake et al. [46], and Tanaka et al. [47, 48] on laboratory mice clearly supported our hypothesis that males eliminated Hg at a substantially faster rate than females in higher vertebrates. These researchers provided compelling evidence that testosterone enhanced Hg-elimination rate in males. Because testosterone is found in teleost fishes as well as higher vertebrates [51–54], the higher Hg-elimination rate in males is a characteristic that is expected to be shared by all of these vertebrates. Moreover, γ-GTP is ubiquitous and conserved in all forms of life [77]. Thus, all of these vertebrates possess γ-GTP, which is the enzyme believed to be involved with the enhancement of Hg-elimination rate in male laboratory mice [46, 47]. Testosterone has been shown to increase the activity of γ-GTP in the kidneys of laboratory mice [46, 47].

## Future research directions

We recommend that fish populations continue to be surveyed to better assess the prevalence of the characteristic of males exceeding females in whole-fish PCB concentration. We contend that this characteristic is primarily driven by a higher rate of energy expenditure in males, although the growth dilution effect can make a substantial contribution in some cases. To date, this characteristic has been documented in eight fish species representing the families of Percidae, Salmonidae, Gadidae, Petromyzontidae, and Paralichthyidae, and the orders of Perciformes, Salmoniformes, Gadiformes, Petromyzontiformes, and Pleuronectiformes. Many families and orders of fishes remain to be surveyed [40].

To better assess the prevalence of a higher rate of energy expenditure in males compared with females in fish populations, we recommend that (1) when feasible, telemetry be used to quantify the sex difference in swimming activities, and (2) laboratory respirometry be used to quantify the sex difference in SMRs. Movements of fish can be tracked with both active and passive telemetry, using acoustic tags attached to fish [78–81]. We point out, however, that movements of fish would have to be tracked at a fine geographic scale to accurately quantify the sex difference in swimming activities, because such quantification depends on accurate estimates of swimming speed and/or total distance traveled over time. Alternatively, the sex difference in swimming activities may be assessed via use of accelerometer tags [82, 83], or use of heart rate telemetry and tailbeat telemetry [11, 12].

Eventually, sex-specific fish bioenergetics models can be developed based on the assessments of sex differences in swimming activities and SMRs as well as the sex differences in PCB concentrations. Fish bioenergetics models have proven invaluable in assessing the strength of the predator-prey trophic link in ecosystems, guiding management of important fisheries, quantifying the effects of various factors on fish growth and consumption, predicting changes in fish growth and consumption with climate change, and assessing the role of fish in cycling nutrients in aquatic ecosystems [84, 85]. For many fish bioenergetics model applications, sex-specific information is not required. Nevertheless, sex-specific bioenergetics models may be appropriate for certain applications [4, 86]. Presently, sex-specific fish bioenergetics models are not available [87, 88]. As previously mentioned, the ratio of whole-fish PCB concentration in males to whole-fish PCB concentration in females should equal the ratio of GGE for females to GGE for males, barring the possibility of a hot spot effect. Thus, we propose that the sex difference in whole-fish PCB concentrations can be used to evaluate the performance of sex-specific fish bioenergetics models. By applying the sex-specific bioenergetics models to female and male fish from a population, estimates of cumulative GGE for both females and males can be generated. Then, the ratio of GGE for females to GGE for males can be compared with the ratio of whole-fish PCB concentration in males to whole-fish PCB concentration in females to check on the accuracy of the sex-specific models’ estimates of food consumption; the ratios should be equal provided the food consumption estimates are accurate. The ratio of GGE for females to GGE for males is determined by the combined effects of the sex difference in energy expenditure rates and growth dilution. As previously explained, the growth dilution effect can be an important determinant of this ratio in some cases, whereas the effect of growth dilution on this ratio can be negligible in other cases. PCB ratios listed in Table 1 can be adjusted by removing the contribution of the growth dilution effect, and we propose that this adjusted ratio is equivalent to the ratio of energy expenditure rate in males to energy expenditure rate in females. These adjusted ratios range from 1.16 to 1.39, indicating that males expended energy at a rate between 16 and 39 % higher than that for females, depending on the fish species.

We also recommend that fish populations continue to be surveyed to better assess the pervasiveness of the characteristic of the ratio of whole-fish PCB concentration in males to whole-fish PCB concentration in females exceeding the ratio of whole-fish Hg concentration in males to whole-fish Hg concentration in females. We have ascribed this characteristic to certain androgens enhancing Hg-elimination rate in males of teleost fishes. In contrast, agnathan fishes (e.g., sea lamprey) appear to lack the androgens needed to enhance Hg-elimination rate in males. To date, four species of teleost fishes and one species of agnathan fishes have been surveyed. Determining PCB and Hg concentrations in both sexes of fishes not considered as primitive as agnathan fishes but more primitive than teleost fishes may be quite instructive. For example, both bowfin (*Amia calva*) and American paddlefish (*Polyodon spathula*) are considered more evolutionarily advanced than agnathans but not as evolutionarily advanced as teleosts [40]. The question of whether Hg-elimination rate is enhanced in males compared with females in fishes like bowfin and American paddlefish remains unanswered.

Based on our synthesis findings, interspecific variation in sex differences in Hg-elimination rates of fishes needs to be elucidated. To the best of our knowledge, only one study has directly addressed the sex difference in Hg-elimination rates, and results showed that male northern pike eliminated Hg at more than double the rate of that for female northern pike. However, the interspecific variability in the differences between the PCB ratio and the Hg ratio listed in Table 1 suggested that the sex difference in Hg-elimination rates varied substantially from one fish species to another. This interspecific variation in the sex differences in Hg-elimination rates must be quantified if we are to make any headway in understanding the mechanisms responsible for the interspecific variation in the differences between the PCB ratio and the Hg ratio.

Once sex differences in Hg-elimination rates have been quantified across a variety of fish species, mass balance models for Hg body burden in fish can be refined. The use of Hg mass balance models to estimate food consumption by fish has rapidly increased since 2000 [15, 16, 89, 90]. The Hg elimination submodel used in these Hg mass balance models was based on a regression model developed by Trudel and Rasmussen [41]. These researchers gleaned the published literature for estimates of rates at which fish eliminate Hg, and then they fitted a regression model with Hg-elimination rate as the dependent variable and fish weight and water temperature as the independent variables. Hg-elimination rate did not vary with fish species or sex in the regression model. Given the available evidence, Hg-elimination rate not only appears to substantially vary between the sexes but Hg-elimination rate of males also appears to vary substantially from one fish species to another. In addition, the rates of Hg elimination by fish may be slower than those portrayed in the Hg elimination submodel based on recent research. Results from a set of whole-lake manipulation experiments involving isotopically enriched Hg suggested that the Hg elimination submodel overestimated Hg-elimination rates for yellow perch and northern pike by 49 to 209 %, depending on whether the chronic exposure form or the acute exposure form of the Hg elimination submodel was used [42, 43]. In sum, the Hg mass balance models may need further refinement to account for species-specific Hg-elimination rates. When sex-specific applications are required, sex-specific Hg-elimination rates will be needed to account for the apparent disparity in Hg-elimination rates between the sexes.

Although Yasutake et al. [46] have proposed a mechanism by which testosterone enhances Hg-elimination rate in male laboratory mice, much research work remains to fully elucidate the molecular and cellular pathways involved with the acceleration of the rate at which Hg is eliminated from the bodies of teleost fishes and higher vertebrates by certain androgens. For example, to the best of our knowledge, the role of the kidneys in eliminating Hg from the bodies of teleost fishes is not well understood. All vertebrates have kidneys, but kidney function varies somewhat from one class of vertebrates to another [91]. Kidneys play a role in the regulation of solute and water composition of the internal environment of all vertebrates. In fish, gills also contribute toward the regulation of solute and water composition within the internal environment. Only in mammals is regulation of the solute and water composition of the internal environment solely dependent on kidney function [91]. Once Hg has been assimilated into the bodies of mammals, the predominant pathway for Hg elimination appears to be urine excretion [45]. In fish, some Hg may potentially be eliminated via excretion from the gills, although the relative importance of kidneys versus gills in eliminating Hg is poorly understood, based on our literature review. In addition, the roles of kidneys and gills in Hg elimination may vary between marine and freshwater fish, because kidneys and gills function to eliminate excess salts and conserve water in marine fish, whereas kidneys and gills function to eliminate excess water and conserve salts in freshwater fish [91]. Much more research is needed to reveal the molecular and cellular mechanisms by which androgens like testosterone and 11-ketotestosterone apparently enhance Hg-elimination rate in male teleost fishes. Moreover, additional research is required to identify the reasons for the apparent ineffectiveness of the androgens found in sea lamprey in enhancing Hg-elimination rate in male sea lamprey. This line of new research could eventually lead to a more effective antidotal protocol for Hg intoxication in humans and other vertebrates.

To assess the effect of GSI of males on testosterone and 11-ketotestosterone concentrations in male fishes, concentrations of these androgens in males, as well as GSIs of males, would need to be determined in a variety of fish species. Regression analyses could then be performed to evaluate the importance of the GSI effect on androgen concentrations. For the most reliable results, methods to determine androgen concentrations should be standardized. Typically, radioimmunoassay (RIA) has been used to determine sex steroid levels in vertebrates. Further, RIA has been conducted with or without extraction, where extraction refers to a technique to isolate the targeted steroids from some of the associated non-target chemicals within the matrix [92, 93]. In cases where extraction is not used, matrix interference may substantially inflate measurements of sex steroid levels [94]. In addition, the resolution of the RIA approach may not be sufficiently fine to accurately measure sex steroid levels, thereby also causing inflated measurements of these levels. At present, the recommended approach for accurately determining androgen levels is to use extraction and chromatography followed by mass spectrometry [94]. To further ensure standardization across fish species, androgen levels should be measured during both the spawning season and the non-spawning season.

Several researchers have advocated that sex differences be specifically incorporated into the assessment of risk to humans and wildlife exposed to contaminants [95–97], and our synthesis findings represented a significant step toward that goal. Sex differences have largely been ignored in risk assessment studies for humans and wildlife exposed to contaminants. Our synthesis results indicated clear differences in contaminant accumulation between the sexes. Males tended to ingest contaminants at a higher rate than females, owing to higher rate of energy expenditure. Consequently, PCB concentrations were higher in males than in females. Our synthesis results also indicated that males appeared to eliminate Hg at a faster rate than females, and that Hg-elimination rates for males appeared to vary substantially from one species to another. Our synthesis findings could be directly applied to existing risk assessment models so that the models could be crudely adjusted to account for sex differences. The models could be further refined as differences between the sexes are further elucidated.

Physiologically based pharmacokinetic (PBPK) modeling has been used to predict Hg concentrations in various tissues and organs of humans and other animal species exposed to Hg over time [98]. Thus, PBPK modeling has provided a valuable tool for assessing risk to humans from exposure to Hg under various environmental scenarios. At present, sex differences are rarely taken into account when performing PBPK modeling of Hg concentrations in humans and animals [98]. Yet, Hg storage sites within the bodies of vertebrates can vary substantially between the sexes [45, 62]. For example, females tend to have higher liver Hg concentrations than males, whereas males tend to have higher kidney Hg concentrations than females. Further, we have assembled a body of compelling evidence for a substantially higher rate of Hg elimination by males than that by females in our synthesis. Consequences of ignoring this sex difference in Hg-elimination rates might include consistent overestimation of Hg concentrations in males and/or consistent underestimation of Hg concentrations in females. A considerable amount of research is still needed to generate a sufficient amount of sex-specific details for these PBPK models so that the accuracy of model predictions can be improved.

## Conclusions

A comparison of the ratio of whole-fish PCB concentration in males to whole-fish PCB concentration in females with the ratio of whole-fish Hg concentration in males to whole-fish Hg concentration in females, both within and between fish species, has led to new insights into sex differences in behavior and physiology of fishes. We submit that these sex differences pertain to higher vertebrates as well. Whole-fish PCB concentration of males consistently exceeded that of females. We conclude that the most plausible explanation for this sex difference was that males expended energy at a higher rate, via both a greater SMR and greater swimming activity, than females. Consequently, males consumed food at a higher rate than females, and therefore, males accumulated PCBs at a higher rate than females. In some cases, the growth dilution effect also contributed to the sex difference in PCB concentrations, but a higher energy expenditure rate for males was still the primary driver of the sex difference. Except in the case of the most primitive fish species included in our synthesis, the ratio of whole-fish PCB concentration in males to whole-fish PCB concentration in females consistently exceeded the ratio of whole-fish Hg concentration in males to whole-fish Hg concentration in females. For sea lamprey, the two ratios were nearly equal. We concluded that the most likely explanation for this pattern was that male teleost fishes eliminated Hg at a faster rate than female teleost fishes, whereas long-term PCB elimination rates were negligible for both sexes and therefore these rates did not vary between the sexes. The faster Hg-elimination rate in males compared with females was most likely attributable to enhancement of the Hg-elimination rate by certain androgens, such as testosterone and 11-ketotestosterone. The sea lamprey does not possess either of these androgens. Thus, the most plausible explanation for the two ratios being nearly equal for sea lamprey was that the androgens found in male sea lamprey do not accelerate Hg-elimination rate, and therefore, the Hg-elimination rates did not vary between the sexes. Additional comparisons of whole-fish contaminant concentrations between the sexes should continue to provide further insights into sex differences in behavior and physiology. Our synthesis findings will eventually contribute toward (1) the development of sex-specific bioenergetics models for fish, (2) the development of sex-specific risk assessment models for humans and wildlife exposed to contaminants, and (3) the refinement of Hg mass balance models for fish and higher vertebrates.

## Abbreviations

CBA/J: A strain of laboratory mice
CFW: A strain of laboratory mice
DEE: Daily energy expenditure
df: Degrees of freedom
GGE: Gross growth efficiency
GSI: Gonadosomatic index
GTP: Glutamyltranspeptidase
Hg: Total mercury
*P*: Attained significance level
PBPK: Physiologically based pharmacokinetic
PCB: Polychlorinated biphenyl
RIA: Radioimmunoassay
SMR: Standard metabolic rate (also known as resting metabolic rate)
*t*: *t* statistic
♀: Female
♂: Male
